# Utility of Readily Available Sugammadex for Emergent Neuromuscular Blockade Reversal

**DOI:** 10.7759/cureus.85932

**Published:** 2025-06-13

**Authors:** Theodore Renner, Kevin Varner, Austin Shaffer, Elizabeth Johnson-Gray, Noah Le, Matthias Franzen

**Affiliations:** 1 Anesthesiology, Ohio University Heritage College of Osteopathic Medicine, Columbus, USA; 2 Anesthesiology, OhioHealth Doctors Hospital, Columbus, USA

**Keywords:** can't intubate can't ventilate, neuromuscular blockade reversal, quality improvement and patient safety, scenario-based simulation, sugammadex

## Abstract

Introduction: Anesthesia providers are often called upon to assist with other providers during can’t intubate, can’t ventilate (CICV) scenarios. The current availability of doses of sugammadex sufficient to provide emergent reversal of neuromuscular blockade in an operating room environment requires visits to multiple operating rooms at our institution. The Anesthesiology Department at OhioHealth Doctors Hospital sought to evaluate the practicality of administering a rescue dose of sugammadex (16 mg/kg) in a CICV scenario. The current storage of sugammadex is six 200 mg vials maximum per operating room, which would provide insufficient dosing for emergent reversal dosing for most adult patients. Furthermore, an audit of our operating room environment found that the average available during a given workday is four vials of sugammadex (800 mg total) per operating room. A simulation-based quality improvement project was devised to test the feasibility of the creation of an emergent reversal kit with six 500 mg vials and a 30 mL syringe in an easily accessible location equidistant from all operating rooms to provide emergent reversal in a CICV scenario.

Methods: The study involved anesthesia trainees (residents and student registered nurse anesthetists (SRNAs), n = 6) participating in a simulated CICV scenario at OhioHealth Doctors Hospital in Columbus, OH, where they were instructed to obtain an emergent reversal dose of sugammadex while others completed the ASA difficult airway algorithm. Each participant completed three iterations of the current arrangement in which they visited multiple operating rooms to obtain the appropriate dose. Participants then completed two iterations in which they utilized an emergent reversal kit. The time from request for sugammadex to the administration of a complete reversal dose was recorded. The data collected were evaluated to determine if a statistically significant improvement in the utilization of the emergent reversal kit was present. A one-tailed paired t-test was used to evaluate if the use of an emergent reversal kit resulted in a statistically significant reduction in the time to administration (p < 0.001).

Conclusion: Our study found a statistically significant decrease in the time to reversal when an emergent reversal kit of six 500 mg vials is placed in an easily accessible location. The mean time for anesthesia residents to successfully reverse neuromuscular blockade was 7.7 minutes on their first attempt without the emergent reversal kit and decreased to 2.9 minutes after practice (on the third attempt). However, implementation of an emergent reversal kit resulted in further time reduction to a mean time of 2.1 minutes on their first attempt and 1.4 minutes on their second attempt when using the emergency reversal kit. These findings reinforce the importance of medication accessibility in high-stakes scenarios and provide a novel method for improving emergency preparedness in anesthesia practice.

## Introduction

Sugammadex is a selective relaxant binding agent that revolutionized the management of neuromuscular blockade induced by aminosteroid neuromuscular blocking drugs such as rocuronium and vecuronium. It encapsulates these agents and assists in the rapid reversal of neuromuscular blockade, making this an important tool in anesthesia [1]. Sugammadex is not only used for routine surgical cases but also plays a significant role in emergencies by offering quick reversal of paralysis that can have an influence on positive patient outcomes. One such scenario is the "can't intubate, can't ventilate" (CICV) situation, a rare but life-threatening emergency in anesthesia [2].

The CICV scenario represents one of the most critical challenges in anesthesia, with only a short window of time to secure a patient's airway and prevent a catastrophic outcome. The ability to quickly reverse paralysis in such patients can induce natural breathing and may avoid the need for invasive airway interventions such as surgical cricothyrotomy [3]. While numerous guidelines emphasize systematic approaches in the management of CICV scenarios, the success of these protocols relies heavily on the quick availability of necessary resources. Despite its proven efficacy, the availability of rescue dose sugammadex (16 mg/kg) is often limited by logistical barriers, such as centralized pharmacy storage and retrieval delays in emergency situations. These limitations offer the chance to further enhance the preparedness of healthcare teams dealing with CICV situations by improving access to sugammadex [2].

Correct identification and quick management of CICV scenarios are important competencies for anesthesiology practitioners, including residents, certified nurse anesthetists, and student nurse anesthetists. The Accreditation Council for Graduate Medical Education (ACGME) cites the importance of simulated clinical situations to learn valuable skills useful in clinical practice [4]. Simulation-based training provides a controlled environment to parallel high-stakes emergencies, evaluate decision-making, and refine technical skills without any risk to patients [4]. Previous simulation-based quality improvement studies have demonstrated the learning model’s effectiveness for high-stakes scenario medical training in both the emergency department and the operating room [5,6,7]. 

In response to the limitations of sugammadex accessibility, the Anesthesiology Department at Doctor’s Hospital designed a simulation-based quality improvement project where an emergent reversal kit containing prepared doses of sugammadex (3000 mg total) was stored near all the operating rooms. This intervention aims to streamline the process of accessing sugammadex and improve response times in simulated CICV scenarios. By comparing time-to-administration metrics with and without the emergent reversal kit, we aim to quantify its potential impact on response times and explore its role in improving preparedness for these high-stakes emergencies. By incorporating this simulation into our curricula, we aim to address an important gap in emergency preparedness while continuing to improve educational outcomes for anesthesia learners. Further, the study provides a template for how this protocol might be translated into clinical practice and provides insights into how emergency airway patient safety can be improved.

## Materials and methods

This simulation involved a patient in whom an airway could not be established after induction of anesthesia. The simulations were conducted at OhioHealth Doctors Hospital in Columbus, OH, from June 29, 2023, to August 22, 2023. Participants of the simulation (n = 6) were anesthesia residents or student registered nurse anesthetists (SRNAs). The materials needed for this simulation included a Laerdal SimMan 3G PLUS mannequin, a standard operating room suite with a ventilator and airway supplies, 16 2 mL vials of normal saline, six 5 mL vials of normal saline, and Pyxis MedStation ES systems in their typical locations across four operating rooms. 

To simulate the average amount of sugammadex available in each operating room at any point of the day, only four 200 mg vials filled with the equivalent volume of normal saline (2 mL) were available in each Pyxis machine. The participants also had access to all medical supplies in the central surgical core that connected each of the operating rooms. A total of five iterations of the simulation were conducted by each participant.

On the initial run of the simulation, the participants were called into the operating room with the ongoing simulated CICV scenario taking place and were only instructed to obtain an emergent reversal dose of sugammadex (16 mg/kg). Anesthesia residents and SRNAs were then evaluated for the time to administration of an emergent reversal dose of sugammadex for a 125 kg patient who required a total of 2000 mg of sugammadex (minimum of 10 200 mg vials or four 500 mg vials). The participants were then given two more attempts at the scenario using 200 mg vials dispersed across multiple operating rooms to see how much their time to administer the reversal dose improved after being familiar with the scenario and simulation. 

On the fourth attempt, once the simulation started, the participants were informed that an emergency sugammadex reversal kit was placed in the central surgical core and were asked to retrieve it to use during the simulation. The kit contained six vials, each filled with the equivalent volume of normal saline (5 mL) for a standard 500 mg vial of sugammadex. The participants then completed the simulation using the kit that required a minimum of four 500 mg vials to give the correct 2000 mg reversal dose. This scenario was then repeated to see how the participant’s time improved after being familiar with the emergency reversal kit. 

Once the five iterations were completed by each participant, Microsoft Office 365™ Excel (Microsoft Corporation, Redmond, WA, USA) was used to conduct a one-tailed paired t-test to evaluate if the use of an emergency reversal kit resulted in a significant reduction in time to administer a reversal dose of sugammadex.

## Results

A total of five simulation runs were done to assess the impact of an emergent reversal kit on the time to administration of sugammadex in a simulated CICV scenario. The participants included anesthesia residents and SRNAs, who were evaluated on their ability to retrieve and administer a full dose (2000 mg) of sugammadex to a simulated 125 kg patient.

Our study found that the introduction of an emergent reversal kit containing six 500 mg vials of sugammadex significantly reduced the time to administration. Without the kit, the mean time for first-attempt administration was 464 seconds, which decreased to 175 seconds by the third attempt after repeated practice. However, when the emergent reversal kit was available, the mean time for first-attempt administration was 124 seconds, improving further to 87 seconds on the second attempt.

A paired t-test demonstrated a statistically significant reduction in the time to administration with the emergent reversal kit compared to the standard retrieval method (p < 0.001). These findings suggest that improved accessibility to high-dose sugammadex in emergency situations can enhance response times and potentially improve patient outcomes during critical airway emergencies.

Tables 1-2 summarize the progressive reduction in the time to administration across simulation runs, highlighting the impact of both the emergent reversal kit and repeated exposure to the scenario.

**Table 1:** Time to administer full reversal dose (seconds) Time recorded of anesthesia learners from the request for emergent reversal dose sugammadex (16 mg/kg) of a 125 kg patient to time of administration of the full dose (2000 mg). Runs 1 to 3 demonstrate the current format in which an average of four vials of 200 mg sugammadex each are in an operating room. Runs 4 and 5 demonstrate usage of an easily accessible emergent reversal kit containing six 500 mg vials of sugammadex (3000 mg total).

Participant	Run 1	Run 2	Run 3	Run 4*	Run 5*
1	410	225	166	92	69
2	430	221	190	186	94
3	345	221	204	107	104
4	566	221	171	148	112
5	402	227	170	85	83
6	631	143	147	124	59
Mean (seconds)	464	210	175	124	87
Mean (minutes)	7.7	3.5	2.9	2.1	1.4

**Table 2:** Time difference between simulation runs (seconds) Run 1 minus Run 5 demonstrates the improvement of each participant with a reduction in the time to reversal from the first attempt with the current arrangement of sugammadex availability the final attempt with the use of the emergent reversal kit. Run 1 minus Run 3 demonstrates how practicing the simulation further improves performance even without the emergency reversal kit. Run 3 minus Run 4 demonstrates the difference between use of current arrangement of availability of sugammadex versus the implementation of a sugammadex reversal kit. Run 3 minus Run 5 demonstrates how practice and familiarity with the emergent reversal kit further improves performance. A one-tailed pair t-test was used to calculate the p-values.

Participant	Run 1 minus Run 5	Run 1 minus Run 3	Run 3 minus Run 4	Run 3 minus Run 5
1	341	244	74	97
2	336	240	4	96
3	241	141	97	100
4	454	395	23	59
5	319	232	85	87
6	572	484	23	88
Mean difference (seconds)	377	289	51	88
Mean difference (minutes)	6.3	4.8	0.9	1.5
p-value	0.000265	0.00121	0.0119	0.0000150

## Discussion

In anesthesia, a CICV scenario is a rare but life-threatening airway emergency that demands immediate intervention to prevent severe hypoxia and patient harm. Anesthesia providers are often called upon to manage these critical situations, where sometimes rapid reversal of neuromuscular blockade is required. In these circumstances, rapid reversal can be lifesaving, with cellular injury and permanent brain damage occurring within minutes of severe hypoxia [8]. The introduction of sugammadex has transformed our approach to rapidly reversing neuromuscular blockade. Within minutes, sugammadex can reverse a deep neuromuscular blockade, thus potentially reducing the time spent in hypoxia. A case report-based review of eight reported cases where sugammadex was administered in CICV scenarios showed that the dose given in these scenarios was often less than the recommended reversal dose with a median (range) of 14 (5-16) mg/kg and also that it took a median (range) of six (2-10) minutes to administer any amount sugammadex following rocuronium administration [9]. A vial at our institution contains 200 mg of sugammadex, and on average, each operating room has anywhere from three to five vials in the backstand. However, the dose required for emergent reversal is often larger than what is readily available in each individual operating room, posing logistical challenges that may delay administration during time-sensitive events.

Recovery from deep neuromuscular blockade has already been shown to be faster with a dose of 4 mg/kg sugammadex than neostigmine [2]. In our hospital system, there is no emergency reversal kit and no operational standard on what to do should rapid reversal of neuromuscular blockade be required. Many studies have shown the superiority of sugammadex versus neostigmine [10,11], but no study has shown the efficacy of having an emergency reversal kit available in their institution.

A previous simulation-based study examined the time for anesthesia teams of varying experience to administer the emergent reversal dose of sugammadex at their institution. They found that the more experienced team administered sugammadex in an average of 5.9 minutes versus 7.2 minutes by the less experienced team. In addition, only four out of the 11 teams administered the correct emergency reversal dose of sugammadex [12]. Our study builds upon these findings by demonstrating that the time to administer the reversal dose can improve with practice. When conducting the simulation using our current setup, the time to administer lessened by an average of 4.8 minutes (p = 0.001) even without the emergency reversal kit after practicing the CICV scenario multiple times. This further shows how impactful simulations can be when preparing for emergency situations. 

Our study highlights the additional impact of an emergent reversal kit on response time in a simulated CICV event, demonstrating a significant reduction in the time to administration when the kit was readily available. When anesthesia providers retrieved sugammadex using the standard method, the mean time to administration was 464 seconds (7.7 minutes), improving to 175 seconds (2.9 minutes) after repeated attempts. By contrast, when an emergent reversal kit containing pre-prepared 500 mg vials was available, the mean administration time decreased to 124 seconds (2.1 minutes) on the first attempt and 87 seconds (1.4 minutes) on the second attempt (p < 0.001). These findings reinforce the importance of medication accessibility in high-stakes scenarios and provide a framework for improving emergency preparedness in anesthesia practice.

Despite these promising results, this study has some limitations. The sample size was relatively small, and additional iterations of the simulation will provide more comprehensive data on its effectiveness as a training tool. Moreover, while the emergent reversal kit improved response times in a simulated setting, further studies are needed to evaluate whether similar improvements in clinical practice translate to better patient outcomes. Future research could explore alternative storage solutions, such as dedicated emergency airway carts or automated medication dispensing systems, to further optimize accessibility.

## Conclusions

The creation of an emergency reversal kit tailored for patients who cannot be intubated or ventilated allows providers to rapidly reverse paralysis. This kit helps to ensure patient safety and enhance medical preparedness among the anesthesia staff and operating room staff. This preparedness not only enhances the confidence of the medical team but also helps in streamlining protocol during high-stress situations where every second matters. While there may be challenges related to resource allocation and training requirements, the benefits outweigh the risks when considering patient outcomes during critical emergencies. Ultimately, this study highlights the value of simulation-based quality improvement initiatives and the potential for protocol modifications such as the implementation of an emergent reversal kit to improve response times, streamline crisis management, and enhance patient safety in critical airway emergencies.

## References

[1] Keating GM (2016). Sugammadex: a review of neuromuscular blockade reversal. Drugs.

[2] Hung O, Murphy M (2017). Management of the difficult and failed airway 3rd ed. https://accessanesthesiology.mhmedical.com/book.aspx?bookid=3373.

[3] Natt B, Mosier J (2021). Airway management in the critically ill patient. Curr Anesthesiol Rep.

[4] Accreditation Council for Graduate Medical Education (ACGME) (2022). Program requirements for graduate medical education in anesthesiology. ACGME.

[5] Comp GB, Silver BV, Elliott J, Kalnow A (2020). Utilization of simulation techniques to enhance quality improvement processes in the emergency department. Cureus.

[6] Cain CL, Riess ML, Gettrust L, Novalija J (2014). Malignant hyperthermia crisis: optimizing patient outcomes through simulation and interdisciplinary collaboration. AORN J.

[7] Slakey DP, Simms ER, Rennie KV, Garstka ME, Korndorffer JR Jr (2014). Using simulation to improve root cause analysis of adverse surgical outcomes. Int J Qual Health Care.

[8] Lacerte M, Hays Shapshak A, Mesfin FB (2025). Hypoxic brain injury. StatPearls [Internet].

[9] Abou Nafeh NG, Aouad MT, Khalili AF, Serhan FG, Sokhn AM, Kaddoum RN (2025). Use of sugammadex in "cannot intubate, cannot ventilate" scenarios: a systematic review of case reports. Anesth Analg.

[10] Hristovska AM, Duch P, Allingstrup M, Afshari A (2017). Efficacy and safety of sugammadex versus neostigmine in reversing neuromuscular blockade in adults. Cochrane Database Syst Rev.

[11] Geldner G, Niskanen M, Laurila P (2012). A randomised controlled trial comparing sugammadex and neostigmine at different depths of neuromuscular blockade in patients undergoing laparoscopic surgery. Anaesthesia.

[12] Bisschops MM, Holleman C, Huitink JM (2010). Can sugammadex save a patient in a simulated 'cannot intubate, cannot ventilate' situation?. Anaesthesia.

